# Synthesis and structural data of a Fe-base sodium metaphosphate compound, NaFe(PO_3_)_3_

**DOI:** 10.1016/j.dib.2015.05.022

**Published:** 2015-06-10

**Authors:** Xinghao Lin, Yanming Zhao, Youzhong Dong, Quan Kuang

**Affiliations:** a School of Material Science and Engineering, South China University of Technology, Guangzhou 510640, PR China; b State Key Laboratory of Luminescent Materials and Devices, South China University of Technology, Guangzhou 510640, PR China; c School of Physics, South China University of Technology, Guangzhou 510640, China

## Abstract

This data article contains the synthesis and structure information of a new Fe-base sodium metaphosphate compound, which is related to the research article entitled ‘Synthesis, structural, magnetic and sodium deinsertion/insertion properties of a sodium ferrous metaphosphate, NaFe(PO_3_)_3_’ by Lin et al. [1]. The research article has reported a new Fe-base metaphosphate compound NaFe(PO_3_)_3_, which is discovered during the exploration of the new potential electrode materials for sodium-ion batteries. In this data article, the synthesized process of this metaphosphate compound and the morphology of the obtained sample will be provided. The high-power XRD Rietveld refinement is applied to determine the crystal structure of this metaphosphate compound and the refinement result including the main refinement parameters, atomic coordinate and some important lattace parameters are stored in the cif file. Also, the refined structure has be evaluated by checkcif report and the result is also provided as the supplementary materials.

## Specifications Table

**Table:** 

Subject area	*Chemistry*
More specific subject area	*Crystal chemistry*
Type of data	*Image, figure*
How data was acquired	*SEM* (FE-SEM, Navo NanoSEM430)*, polycrystal-powder XRD (*X’Pert PRO, PANalytical, Netherlands)*,* GSAS program.
Data format	*Raw data.*
Experimental factors	*Determination of a new* Fe-base sodium metaphosphate compound
Experimental features	*Sample was obtained by solid-state method by heating at 600* *°C. The structure is determined by Polycrystal-powder XRD Rietveld refinement.*
Data source location	*South China University of Technology, Guangzhou*
Data accessibility	*The data are supplied with this article*

## Value of the data

• The data provided the base information of the metaphosphate compound NaFe(PO_3_)_3_.
• The data provided the experimental and calculated XRD patterns of NaFe(PO_3_)_3_ compound.
• The data provided the structure refinement result of NaFe(PO_3_)_3_ compound.
• The data also provide the check if report of the refined structure.

## 1 Experimental design, materials and methods

### 1.1 Sample preparation

**Fig. 1:**
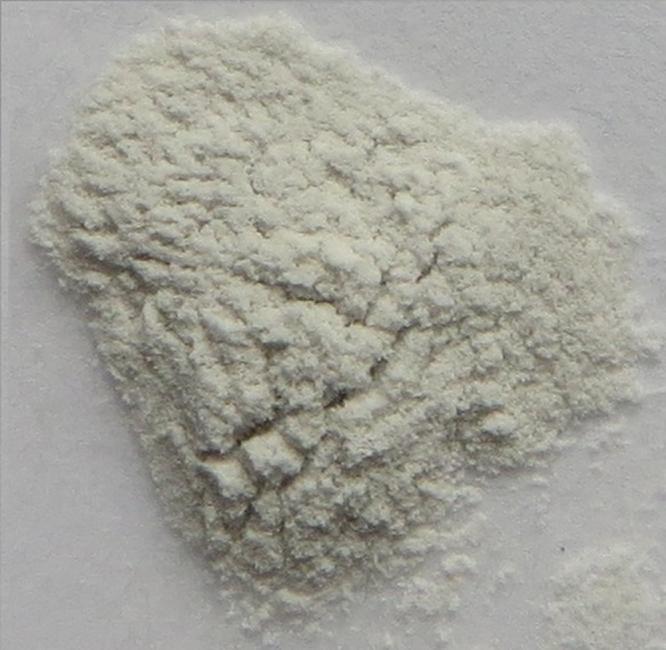
The macroscopic feature of NaFe(PO_3_)_3_ sample obtained by solid state method.

NaFe(PO_3_)_3_ compound was synthesized by conventional two-step solid-state method and Na_2_CO_3_ (Aladdin, ≥99.8%), FeC_2_O_4_·2H_2_O (Aladdin, ≥99.9%) and NH_4_H_2_PO_4_ (Codow, ≥99.9%) powder reagents were used as the raw materials. The stoichiometric proportions of these raw materials with the molar rate of Na:Fe:P=1:1:3 was carefully grand homogeneous in the agate mortar, put into the platinum crucible, and then preheated at 573 K for 10 h to expelling NH_3_, H_2_O and CO_2_. After cooling down to room temperature, the samples were reground again in the agate mortar for 30 min, and then sintered at 873 K for 15 h in Pt crucible. After cooling down to room temperature naturally, the NaFe(PO_3_)_3_ compound was obtained. In order to prevent forming of the trivalent iron, all this sintering process was carried out under the reducing atmosphere (5%H_2_+95%Ar atmosphere). Fig. 1 show photograph of the NaFe(PO_3_)_3_ sample synthesized by solid-state method and as can be observed, the white powder can be obtained.

### 1.2 Morphology of NaFe(PO_3_)_3_

**Fig. 2:**
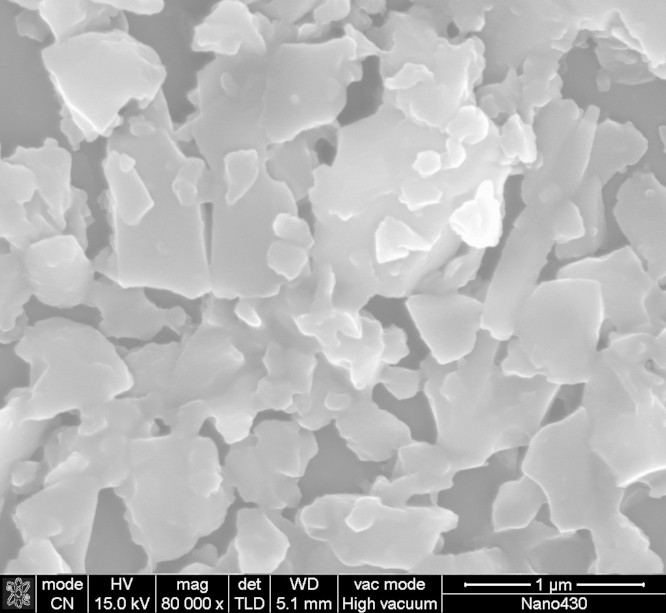
The typical microstructure of NaFe(PO_3_)_3_ sample obtained by solid state method.

NaFe(PO_3_)_3_ powders were dissolved in ethyl alcohol and ultrasound for 30 min to disperse homogeneous. The mixture solutions were then adsorbed in the surface of the mica-sheet and dry in air directly. The typical SEM image of NaFeP_3_O_9_ powder, as shown in Fig. 2, was obtained by the field emission scanning electron microscopy (FE-SEM, Navo NanoSEM430).

### 1.3 Polycrystal-powder XRD characterization

There is no special treatment for the sample before the polycrystal-powder XDR characterization. The sample was only slightly grand in the agate mortar after the sintering process and then the white powder was obtained. The powder diffraction intensity data for the sample were collected using X’Pert PRO (PANalytical, Netherlands) with Cu *Kα* radiation (*λ*=1.5418 Å) and a graphite monochromator was used for diffracted beams. Data were collected by a step scan mode with a scanning step of 0.02° at a sampling time of 3 s. This measurement was carried out at the room temperature.

### 1.4 Structure refinement and determination

**Fig. 3:**
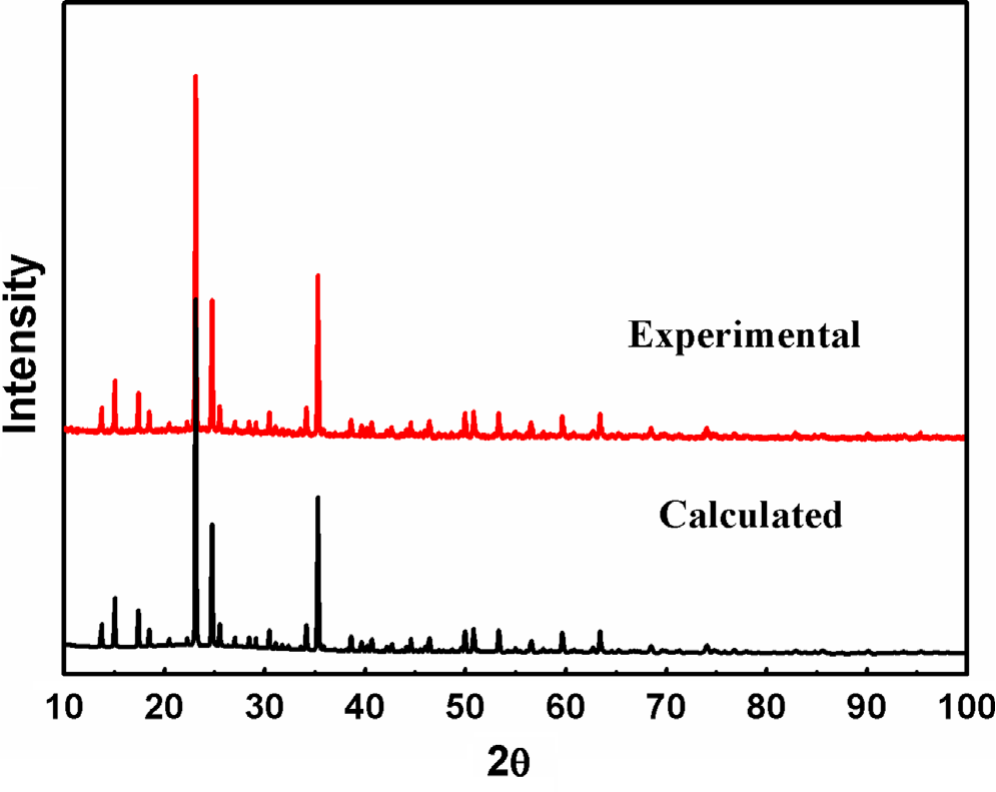
XRD patterns of NaFeP_3_O_9_ compound obtained by the experimental and calculated method, respectively.

**Fig. 4:**
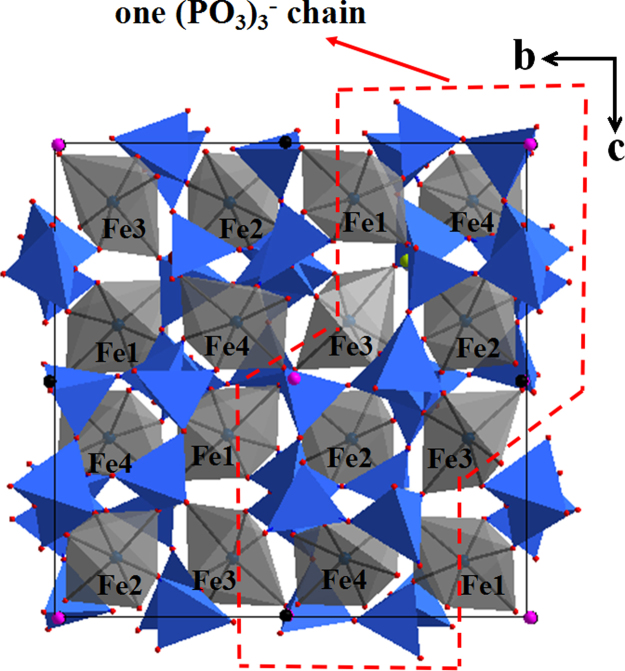
View of the typical NaFe(PO_3_)_3_ structure projected onto the *b*–*c* plane, where PO_4_ tetrahedrons and FeO_6_ octahedrons arranged. The circle part presented as a one-dimensional (PO_3_)^1−^ chain along *a*-axis.

**Fig. 5:**
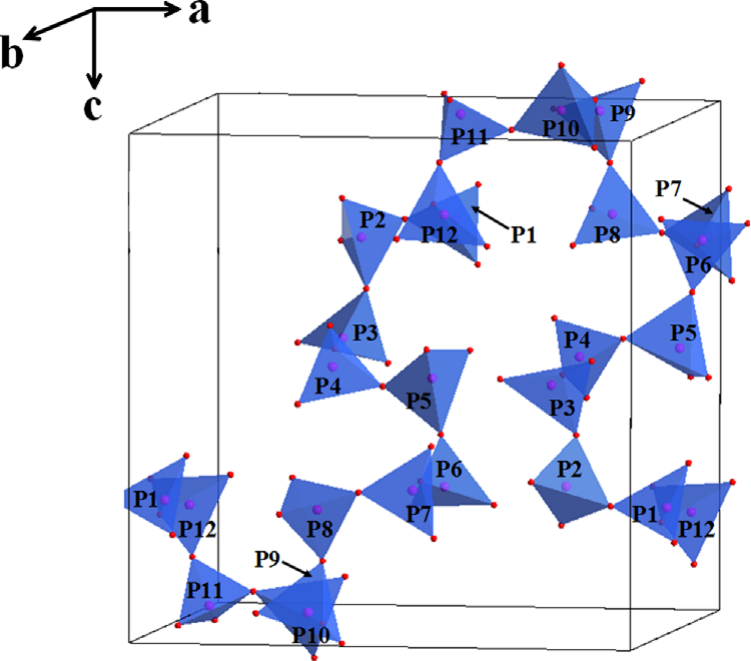
Projection of the one-dimensional (PO_3_)^−^ chain and PO_4_ tetrahedrons have been arranged, which have presented as the middle chain groups connected *via* oxygen atoms along *a*-axis, with two bridging and two terminal oxygen atoms in each tetrahedron.

The structure of NaFe(PO_3_)_3_ compound is determined by the single-phase mode *Rietveld* refinement using the GSAS program [2] *via* the EXPGUI interface [3] and a space group of *P2*_*1*_*2*_*1*_*2*_*1*_ (No. 19) was selected as the refined model. The starting positional parameters of all atoms were taken by analogy with those of the corresponding atoms in its isomorphic structures [4,5]. The calculated XRD pattern produced by GSAS program was compared with that obtained by experimental method, as shown in Fig. 3. The refinment result including the main refinement parameters, atomic coordinate and some important lattace parameters are stored in cif file (provided in Supplementary materials), which is produced by GSAS program. The crystal structure of NaFe(PO_3_)_3_ compound determined by the obtained cif file is shown in Figs. 4 and 5. Also, the obtained cif file is evaluated on the website of http://checkcif.iucr.org/ and its checkcif report is provided as the Supplementary materials.

## Footnotes

Appendix A Supplementary data associated with this article can be found in the online version at doi:10.1016/j.dib.2015.05.022.
