# Supplementary material for: Synthesis and structural data of a Fe-base sodium metaphosphate compound, NaFe(PO3)3
Source: Data Brief. 2015 Jun 10;4:217–21. doi: 10.1016/j.dib.2015.05.022 (PMC4510540; doi:10.1016/j.dib.2015.05.022)

checkCIF/PLATON report

Structure factors have been supplied for datablock(s) NFP-CIF_publ

THIS REPORT IS FOR GUIDANCE ONLY. IF USED AS PART OF A REVIEW PROCEDURE FOR PUBLICATION, IT SHOULD NOT REPLACE THE EXPERTISE OF AN EXPERIENCED CRYSTALLOGRAPHIC REFEREE.

No syntax errors found.   CIF dictionary   Interpreting this report

Datablock: NFP-CIF_publ

```
Bond precision:  P- O = 0.1130 A        Wavelength=1.54060

Cell:             a=14.339(3)     b=14.337(3)     c=14.3642(13)
                  alpha=90        beta=90         gamma=90
Temperature:      298 K

                 Calculated                Reported
Volume           2953.1(9)                 2953.1(3)
Space group      P 21 21 21                P 21 21 21
Hall group       P 2ac 2ab                 ?
Moiety formula   Fe4 O36 P12, 4(Na)        ?
Sum formula      Fe4 Na4 O36 P12           Fe4 Na4 O36 P12
Mr               1263.00                   1263.00
Dx,g cm-3        2.841                     0.000
Z                4                         4
Mu (mm-1)        23.577                    0.000
F000             2464.0                    0.0
F000’            2470.15
h,k,lmax
Nref
Tmin,Tmax
Tmin’

Correction method= Not given

Data completeness=                Theta(max)=

R(reflections)=                   wR2(reflections)=

S =                          Npar=
```

The following ALERTS were generated. Each ALERT has the format
test-name_ALERT_alert-type_alert-level.
Click on the hyperlinks for more details of the test.

Alert level G

```
PLAT004_ALERT_5_G Polymeric Structure Found with Maximum Dimension          3 Info
PLAT152_ALERT_1_G The Supplied and Calc. Volume s.u. Differ by ...          6 Units
PLAT791_ALERT_4_G The Model has Chirality at P3      (Chiral SPGR)          R Verify
PLAT791_ALERT_4_G The Model has Chirality at P4      (Chiral SPGR)          S Verify
PLAT791_ALERT_4_G The Model has Chirality at P7      (Chiral SPGR)          R Verify
PLAT791_ALERT_4_G The Model has Chirality at P8      (Chiral SPGR)          R Verify
PLAT791_ALERT_4_G The Model has Chirality at P10     (Chiral SPGR)          R Verify
PLAT791_ALERT_4_G The Model has Chirality at P11     (Chiral SPGR)          S Verify
PLAT981_ALERT_1_G No non-zero f" Anomalous Scattering Values Found     Please Check
PLAT986_ALERT_1_G No non-zero f' Anomalous Scattering Values Found     Please Check
```

```
   0 ALERT level A = Most likely a serious problem - resolve or explain
   0 ALERT level B = A potentially serious problem, consider carefully
   0 ALERT level C = Check. Ensure it is not caused by an omission or oversight
  10 ALERT level G = General information/check it is not something unexpected

   3 ALERT type 1 CIF construction/syntax error, inconsistent or missing data
   0 ALERT type 2 Indicator that the structure model may be wrong or deficient
   0 ALERT type 3 Indicator that the structure quality may be low
   6 ALERT type 4 Improvement, methodology, query or suggestion
   1 ALERT type 5 Informative message, check
```

It is advisable to attempt to resolve as many as possible of the alerts in all categories. Often the minor alerts point to easily fixed oversights, errors and omissions in your CIF or refinement strategy, so attention to these fine details can be worthwhile. In order to resolve some of the more serious problems it may be necessary to carry out additional measurements or structure refinements. However, the purpose of your study may justify the reported deviations and the more serious of these should normally be commented upon in the discussion or experimental section of a paper or in the "special_details" fields of the CIF. checkCIF was carefully designed to identify outliers and unusual parameters, but every test has its limitations and alerts that are not important in a particular case may appear. Conversely, the absence of alerts does not guarantee there are no aspects of the results needing attention. It is up to the individual to critically assess their own results and, if necessary, seek expert advice.

Publication of your CIF in IUCr journals

A basic structural check has been run on your CIF. These basic checks will be run on all CIFs submitted for publication in IUCr journals (*Acta Crystallographica*, *Journal of Applied Crystallography*, *Journal of Synchrotron Radiation*); however, if you intend to submit to *Acta Crystallographica Section C* or *E*, you should make sure that full publication checks are run on the final version of your CIF prior to submission.

Publication of your CIF in other journals

Please refer to the *Notes for Authors* of the relevant journal for any special instructions relating to CIF submission.

PLATON version of 29/01/2015; check.def file version of 29/01/2015

Datablock NFP-CIF_publ - ellipsoid plot

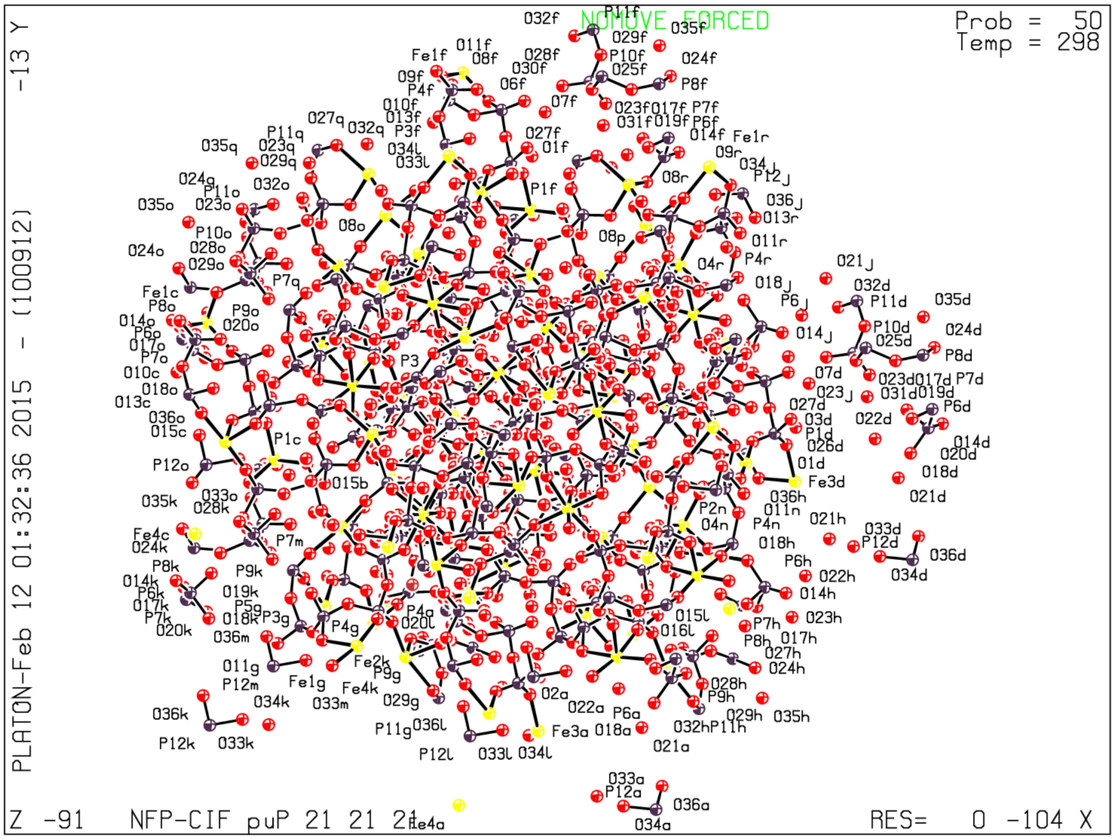

Supplement: Supplementary file 1 — Supplementary data [file mmc1.pdf]
